# Plasmatic coagulation profile after major traumatic injury: a prospective observational study

**DOI:** 10.1007/s00068-022-01971-6

**Published:** 2022-05-16

**Authors:** Michael Caspers, Nadine Schäfer, Bertil Bouillon, Victoria Schaeben, Monica Christine Ciorba, Marc Maegele, Jens Müller, Bernd Pötzsch

**Affiliations:** 1 The Institute for Research in Operative Medicine, Faculty of Health, Department of Medicine, Witten/Herdecke University, Ostmerheimer Str. 200, 51109 Cologne, Germany; 2 Department of Traumatology, Orthopaedic Surgery and Sports Traumatology, Cologne-Merheim Medical Centre (CMMC), Witten/Herdecke University, Campus Cologne-Merheim, Ostmerheimer Str. 200, 51109 Cologne, Germany; 3 Institute of Experimental Haematology and Transfusion Medicine, University Clinic Bonn, Sigmund-Freud-Str.25, 53105 Bonn, Germany

**Keywords:** APC, Thrombin, Trauma-induced coagulopathy, Fibrinolysis

## Abstract

**Purpose:**

Uncontrolled hemorrhage is still the major cause of preventable death after trauma and is aggravated by trauma-induced coagulopathy (TIC). The underlying pathophysiology of TIC is still elusive, but several key effectors such as the thrombin-generation capacity, the protein C (PC) pathway, and the fibrinolytic activity could be identified. The aim of this prospective observational study was to investigate plasma coagulation markers attributed to reflect the course of TIC and to identify the mechanisms being responsible for the coagulopathy after major trauma.

**Methods:**

Seventy-three consecutive patients after major trauma and admission to a level-1-trauma unit were included to the study. During early trauma management, extended coagulation testing including the measurement of circulating thrombin markers and activated PC (APC) was performed and correlated with standard shock parameters and the patients’ clinical course and outcome.

**Results:**

In contrast to standard coagulation parameters, thrombin markers and APC were found to be increased in correlation with injury severity. Even in patients with lower impact mechanisms, early endogenous accumulation of thrombin markers and APC (ISS < 16: 0.5 ng/ml; ISS ≥ 16–26: 1.5 ng/ml; ISS > 26: 4.1 ng/ml) were observed. Furthermore, APC showed ISS- and injury-dependent patterns while ROC curve analysis revealed that especially APC plasma levels were predictive for coagulopathy and general patient outcome.

**Conclusion:**

Increased levels of APC and thrombin markers in patients after major trauma were positively correlated with injury severity. APC showed an ISS- and injury-dependent kinetic and might serve as candidate biomarker to identify patients at risk for developing TIC.

**Supplementary Information:**

The online version contains supplementary material available at 10.1007/s00068-022-01971-6.

## Introduction

Uncontrolled hemorrhage is still the major cause of preventable death after major trauma [1, 2]. About 80% of deaths following uncontrolled bleeding occur within 24 h after trauma. Bleeding is aggravated by the development of trauma-induced coagulopathy (TIC), providing the basis of an intense interest in the understanding of its pathogenesis and the detection of early markers allowing precise diagnosis for more targeted therapy [3, 4].

Research over the last decades revealed TIC as multifactorial clinical entity with different pathways involved. However, whether TIC is a unique clinical entity or part of a disseminated intravascular coagulopathy (DIC), and to identify central key players in the pathophysiological conception remains challenging” [5].

One major concept ascribes the Protein C (PC) pathway a central role in the pathology following traumatic shock and endothelial damage. The PC pathway is induced by a complex formed between thrombin and thrombomodulin (TM) on the endothelial cell surface. Once generated, APC downregulates thrombin formation by inactivation of activated factors V (FVa) and VIII (FVIIIa). Moreover, APC attenuates fibrinolysis by inactivation of plasminogen activator inhibitor-1 (PAI-1). Accordingly, this process is accompanied by the suppression of thrombin generation and an increase in the production of tissue-type plasminogen activator (t-PA), resulting in systemic fibrinolysis [6, 7]. However, there are several contradicting theories, which doubt the central role of activated PC (APC) as a primary agent and, instead, attribute the bleeding phenotype of TIC to massive thrombin formation in conjunction with platelet as well as fibrinogen consumption and fibrinolysis [8–10].

Another current hypothesis focuses on the DIC-fibrinolysis pathway, proposing that the bleeding tendency is secondary to hypoperfusion, shock and endothelial injury. In the further sequelae, this leads to an increased thrombin-generation potential, the consumption of clotting factors and fibrinolysis [11–13].

The aim of the present study is to identify plasmatic factors that are responsible for the hypocoagulative state after trauma and to investigate kinetics of thrombin formation and APC generation in the early phase after major trauma. Plasma levels of active thrombin and APC were measured using oligonucleotide-based enzyme capture assays. In addition, parameters for thrombin-generation and inhibition as well as markers for fibrinolysis were measured and correlated with well-established shock parameters and indicators for coagulopathy.

## Methods

### Recruitment of trauma patients and blood sampling

Between 2017 and 2020, 73 consecutive trauma patients admitted to the emergency department of the Level 1 trauma unit in Cologne-Merheim (Cologne-Merheim Medical Centre, CMMC) who met the following criteria were recruited:

- Age ≥ 18 years;
- No hospital transfer;
- Major trauma defined by the trauma mechanism (e.g., fall from height > 3 m; accidents with pedestrians or (motor-)cyclists, [14]) resulting in an activation of the shock room team according to the recommendations of severely injured in Germany (“Weißbuch DGU^®^”, [15]) and Advanced Trauma Life Support (ATLS)^©^.

**Fig. 1:**
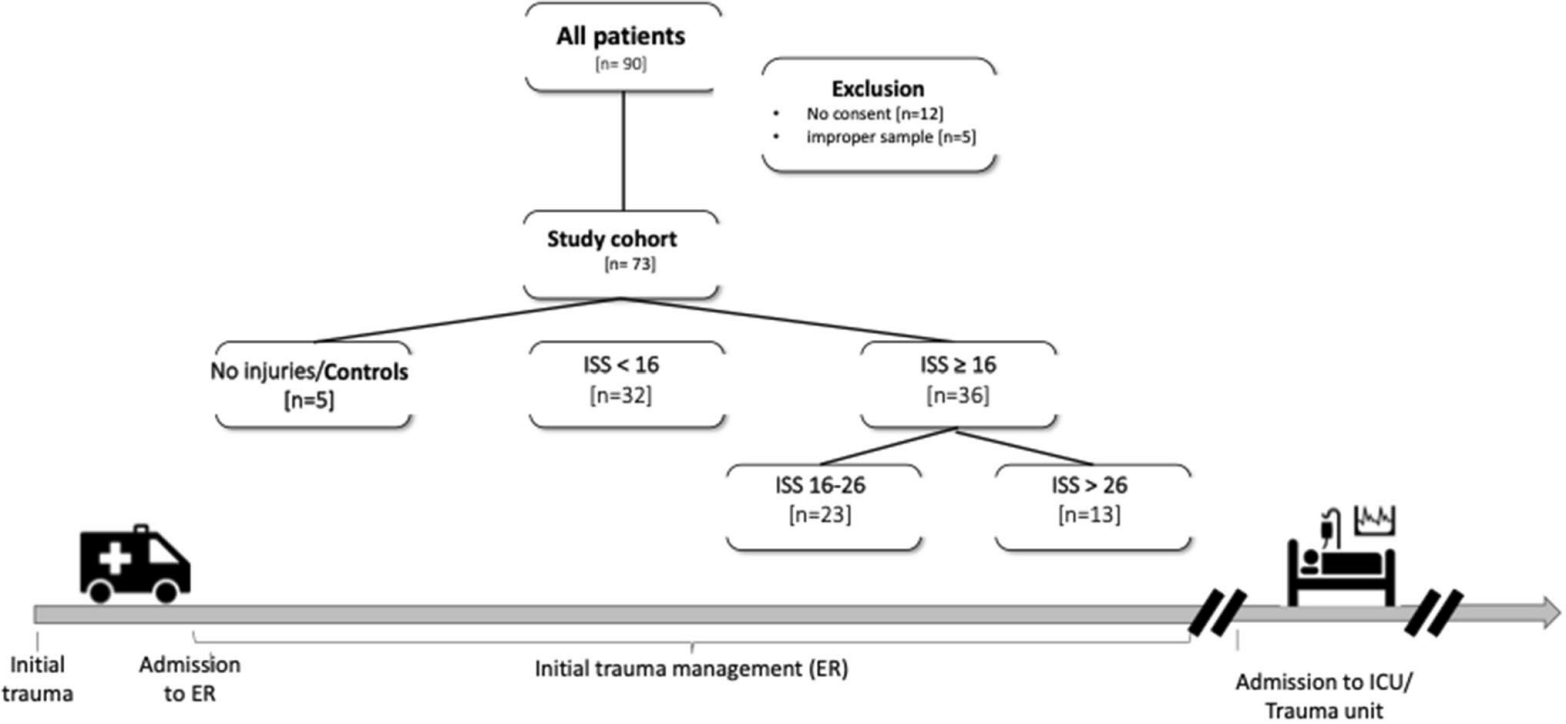
The flow of patients throughout the study according to study groups, exclusion criteria and data sampling

Our recruitment ended after a preassigned period. Characterization of our study cohorts and patients’ course during data collection are summarized in Fig. 1 (c.f. Figure 1).

In accordance with ethical approval (Witten/Herdecke University; 23/2017), patients who were incapable of giving informed consent on admission were included preliminary and following patients’ presumed intent. Written informed consent was obtained as soon as possible during clinical recovery and if not possible, patients were excluded from the study.

Blood samples were drawn during primary survey of the patient within the standardized trauma management via puncture of Arteria or Vena femoralis using a 20G needle (B. Braun, Melsungen, Germany). For preparation of blood collection tubes for plasma APC measurements (APC tubes), Bivalirudin (Angiox^®^, The Medicines Company, Oxfordshire, United Kingdom) and Aprotinin (Applichem, Darmstadt, Germany) were added to a standard citrate monovette^®^ (Sarstedt, Nümbrecht, Germany) to achieve final concentrations of 250 μg/ml (115 μM) and 500 KIU/ml, respectively. For plasma thrombin measurements, Argatroban (Argatra(r), Mitsubishi Pharma, Düsseldorf, Germany) was added to citrate monovettes to achieve a final concentration of 100 µM. Immediately after blood collection, all tubes were centrifuged at 2500×*g* for 15 min, and plasma was aliquoted and stored at − 80 °C until analysis.

Data on blood count (platelets [µl], hematocrit [%], hemoglobin [g/dl]), standard coagulation including aPTT (s), INR, D-Dimer [mg/l], fibrinogen via Clauss method [mg/dl] and clinical chemistry parameters (creatinine [mg/dl], urea [mg/dl], electrolytes [mmol/l], GOT [U/l], GPT [U/l], LDH [U/l], lipase [U/l], lactate [mmol/l], CRP [mg/l])were obtained from the clinical data set. In addition, blood gas analysis was performed for each patient (BGA, GEM 3500, Serial No. 14074200).

As surrogate markers for a clinically relevant bleeding tendency after trauma, we defined:

- A trauma-associated severe hemorrhage (TASH) score ≥ 15 ([16, 17], representing a > 50% risk for massive transfusion), or
- transfusion of blood products with red blood cell concentrates (RBC) within the first 24 h after admission, or
- the clinical judgment of expected bleeding phenotypes within the trauma management based either on thromboelastometry, clinical experience or clinical signs (e.g., prolonged bleeding after punctures, spontaneous or prolonged bleeding).

### Hemostasis biomarkers

Prothrombin activation fragment F1.2 (F1 + 2) and thrombin–antithrombin complexes (TAT) were determined using the Enzygnost TAT micro and F1.2 (monoclonal) ELISA kits (Siemens Healthineers, Marburg, Germany). Plasmin–antiplasmin complexes (PAP) were measured using the TECHNOZYM^®^ PAP Complex ELISA Kit (Technoclone, Vienna, Austria).

For measuring plasma levels of active APC and thrombin, oligonucleotide-based enzyme capture assays (OECA) were applied as previously described [18, 19]. These assays correspond to the commercially available Oligobind^®^ Thrombin and APC assays (Loxo, Dossenheim, Germany).

### Statistical analysis

Statistical analysis was performed using GraphPad Prism version 7.00 (GraphPad Software, La Jolla California USA) and SPSS statistics version 21 (IBM Corp., Armonk, NY). Data are presented as median ± interquartile range (IQR) or the 0.95 confidence interval for continuous variables and as frequency (%) for categorical variables. The Student’s *t* test and Mann–Whitney *U* test were used to compare continuous variables with normal and non-normal distributions, respectively. Furthermore, laboratory data were correlated with clinical data using Spearman’s rank correlation. Receiver-operating characteristic (ROC) curve analysis was conducted calculating area under the curve (AUC) for coagulation markers that could discriminate between coagulopathic and non-coagulopathic patients upon admission. To calculate the approximate cutoff values, the Euclidean distance was minimized between the ROC curve and the top left edge of the diagram.

To identify the influence of the now dichotomized variables on outcome parameters and Sepsis-related Organ Failure Assessment (SOFA) score, a simple logistic regression was applied, respectively. Results were considered as significant if *p *values were *p *≤ 0.05.

## Results

### Patient characteristics

Between 2017 and 2020, a total of 90 trauma patients were recruited to the study, of whom 17 patients had to be excluded, either due to missing informed consent (12 patients) or improper sample preparation (5 patients). Thus, 73 trauma patients were included for further analysis of which 65 suffered a blunt and 8 a penetrating trauma. Four patients additionally had a significant burn injury. Almost half of the patients (*n = *36) sustained serious injuries with an injury severity score (ISS) ≥ 16 and with a median ISS of 25 (cf. Table 1). Among these, a sub-cohort of 13 patients had severe injuries (ISS > 26, median ISS of 38). Forty-four percent of patients (*n = *32) suffered from minor injuries (ISS < 16, median ISS of 5), and 5 patients turned out to be uninjured (controls; cf. Table 1).

**Table 1:** Injury and bleeding characteristics of trauma patients (*n = *73)

Cohort characteristics	
Male sex [a]; *n*	54
Age, median, (IQR)	47 (33.75)
Blunt trauma, *n*	65
28 d mortality, *n*	4
Injury pattern	
Not injured (controls), *n*	5
Minor injured (ISS < 16), *n*	32
Severely Injured (ISS ≥ 16), *n*	36
AISAbdomen ≥ 3, *n*	6
AISThorax ≥ 3, *n*	18
AISHead/neck ≥ 3, *n*	21
AISExtremity ≥ 3, *n*	10
ISS	
Minor injured (ISS < 16), median, (IQR)	5 (5)
Severely injured (ISS ≥ 16), median, (IQR)	25 (5.5)
Severely injured with ISS > 26, median, (IQR)	38 (9)
Signs of coagulopathy	
TASH ≥ 15 *n*; median, (min; max)	10; 17.5 (15; 19)
Transfusion < 24 h *n*; median, (min; max)	22; 2.5 (2;15)
Clinical signs of coagulopathy, *n*	18

Characteristics of our polytrauma cohort separated in injury pattern, injury severity score (ISS) and signs of coagulopathy. As surrogate for coagulopathic patients, the TASH-Score, transfusion of RBCs < 24 h and a clinical evaluation were assumed

Within the group of patients with an ISS ≥ 16, encoded with an abbreviated injury scale (AIS) ≥ 3, injuries of the head were most frequent (*n = *21; 58%)), followed by injuries of the thoracic region (*n = *18; 50%) and those of the extremities (*n = *10; 28%). Six (17%) of the recruited patients with an ISS ≥ 16 had an abdominal trauma (cf. Table 1).

In all groups, minimal intravenous fluid was administered prehospitally in comparable volumes (mean of 620 ml). No patient received artificial colloid or vasoactive agents or blood products prior to blood sampling. Patients with higher injury severity had a significantly longer prehospital rescue time (“time on the road”), as well as ICU and hospital stay and required a ventilator for a longer period (cf. Table 2).

**Table 2:** Characteristics of trauma management

	ISS < 16	ISS ≥ 16–26*	ISS > 26**	*p* value
Time on the road [min], median (IQR)	57.5 (15)	76 (30)	70 (33)	0.02*; 0.05**
Days in hospital, median (IQR)	8 (4)	19 (21)	38 (57)	0.07; < 0.01**
Days in ICU, median (IQR)	3 (1)	7 (10)	19 (32)	< 0.01*; < 0.01**
Ventilation hours [h], median (IQR)	0	6 (78)	121 (681)	< 0.01*; < 0.01**

Values are presented as median for the respective group

*p* values are relative to the ISS < 16 group and marked with (*) for the ISS ≥ 16–26 group and (**) for the ISS > 26 group

Twenty-two patients (30.1%) received blood products within 24 h (any types) of which 8 patients needed to be massively transfused (≥ 10 units of RBCs within 24 h) (cf. Table 1). A TASH score of ≥ 15 had been calculated for 10 patients (13.7%) and 24.7% of patients (*n = *18) were categorized by clinical judgements as “showing signs of coagulopathy”.

### Physiologic characteristics and basic coagulation profiles on admission

All patients within this study were taken to hospital accompanied by an emergency physician either via ground-based rescue service or helicopter transport. At hospital admission, the groups did not differ significantly in terms of external factors that influence coagulation capacity such as temperature, acidosis or relevant hemodilution (cf. Table 3). The sub-cohort with an ISS > 26 significantly more frequently showed clinical signs of shock as represented by a shock index ≥ 1. As expected, the surrogate for tissue damage, Creatinine kinase (CK) increased with injury severity (cf. Table 3). Interestingly, all patients showed normal ranged standard global coagulation parameters (aPTT, INR), regardless of the underlying injury pattern. Only those patients with an ISS > 26 had significantly higher (still normal ranged) INR values (Table 3 and Fig. 1A, B). However, ISS-dependent consumption of coagulation factors was reflected by decreasing levels of functional factor V (FV) and fibrinogen plasma levels (Table 3).

**Table 3:** Physiologic characteristics and coagulation profile of patients on admission

	ISS < 16	ISS ≥ 16–26*	ISS > 26**	*p* value
Temperature [°C]	36.6	35.9	35.5	
Lactate [mmol/l]	1.6 (1.3)	2 (1.4)	4.3 (4.6)	0.26*; 0.05**
Base deficit [mmol/l]	0.7 (4.05)	− 1.05 (3.5)	− 4.4 (12)	0.17*; 0.11**
pCO_2_ [mmHg]	43 (10)	48.5 (6)	50 (12)	0.06*; 0.15**
First blood pressure [mmHg]	130 (21)	120 (20)	100 (60)	0.06*; 0.07**
First heart rate [bpm]	87 (21)	78 (21)	100 (65)	0.11*; 0.07**
Shock index	0.65 (0.2)	0.66 (0.18)	1 (0.54)	0.81*; 0.03**
Hemoglobin [g/dl]	13 (2.1)	12.6 (2)	12 (5)	0.64*; 0.07**
Hematokrit [%]	37,6 (6.5)	38 (6)	34 (14)	0.63*; 0.10**
CK [U/l]	168 (377)	323 (243)	528 (755)	0.07*; 0.002**
Platelets [/nl]	215 (86.3)	243 (101)	224 (130)	0.14*; 0.46**
INR	1.02 (0.08)	1.04 (0.1)	1.12 (0.3)	0.62*; < 0.01**
aPTT [s]	26.7 (7.1)	26.8 (3)	27.5 (10)	0.82*; 0.30**
D-dimer [mg/l]	2 (6.1)	15 (24.2)	35 (11)	< 0.01*; < 0.01**
Fibrinogen [mg/dl]	238 (82.3)	208 (117)	190 (113)	0.3*; < 0.01**
Thrombin [ng/ml]	0.19 (0.41)	0.62 (1.17)	0.67 (1.32)	0.01* 0.01**
APC [ng/ml]	0.5 (1.08)	1.5 (2.67)	4.1 (7.3)	0.02*; < 0.01**
TAT [ng/ml]	31.15 (98.9)	144 (325)	443 (761)	< 0.01*; < 0.01**
F1.2 [nmol/l]	0.703 (0.59)	1.8 (2.44)	8.5 (13.8)	< 0.01* < 0.01**
PAP [ng/ml]	611.4 (1000.7)	1924 (3376)	2221 (4518)	< 0.01*; < 0.01**
PC-activity [%]	90.8 (31.45)	88 (23)	80 (21)	0.18*; < 0.01**
Free PS AG [%]	87.8 (24.92)	86 (17)	76 (38)	0.73*; 0.02**
FV [%]	78.7 (26.85)	68 (44)	56 (28)	0.03*; < 0.01**

All values are presented as median and interquartile range (IQR) in brackets for the respective group

*P* values are relative to the ISS < 16 group and marked with (*) for the ISS ≥ 16–26 group and (**) for the ISS > 26 group

### Dependence of plasma levels of hemostatic biomarkers on injury severity and shock parameters

Thrombin metabolism as reflected by the indirect markers F1 + 2 (thrombin generation) and TAT (thrombin inhibition) was found to be positively correlated with ISS-defined injury severity (Table 3 and Fig. 1C and E). The same was true for APC plasma levels (Table 3 and Fig. 1F). In contrast, plasma levels of active thrombin showed a plateau at ISS ≥ 16 (Table 3 and Fig. 1D). In accordance to the observed activation of the pro- and anticoagulant mechanisms of the coagulation cascade, also plasma levels of PAP and D-Dimer, as markers of fibrinolysis, showed corresponding effects in dependence of underlying ISS values (Table 3 and Fig. 1G and H). Comparing the assessed direct coagulation biomarkers (thrombin and APC) to established shock parameters, a positive correlation of plasma APC levels with the shock index (*r* = 0.36, *p* = 0.005) and serum lactate levels (*r *= 0.504, *p* < 0.0001) was observed. According to ATLS classification of shock, a significant difference in APC plasma levels between lower (class I and II) and higher classes (class III and IV, *p* = 0.015) could be detected (Suppl. Fig. 7).

Regarding plasma thrombin levels, however, no significant correlation of thrombin values to the shock index (*r* = 0.062; *p *= 0.64) or serum lactate (*r *= − 0.128; *p* = 0.366) could be found. Even ATLS shock classes III and IV did not result in significantly higher thrombin values compared to lower classes I and II (*p* = 0.451) (Suppl. Fig. 8).

### Effect of injury pattern on plasma levels of hemostatic biomarkers

**Fig. 2:**
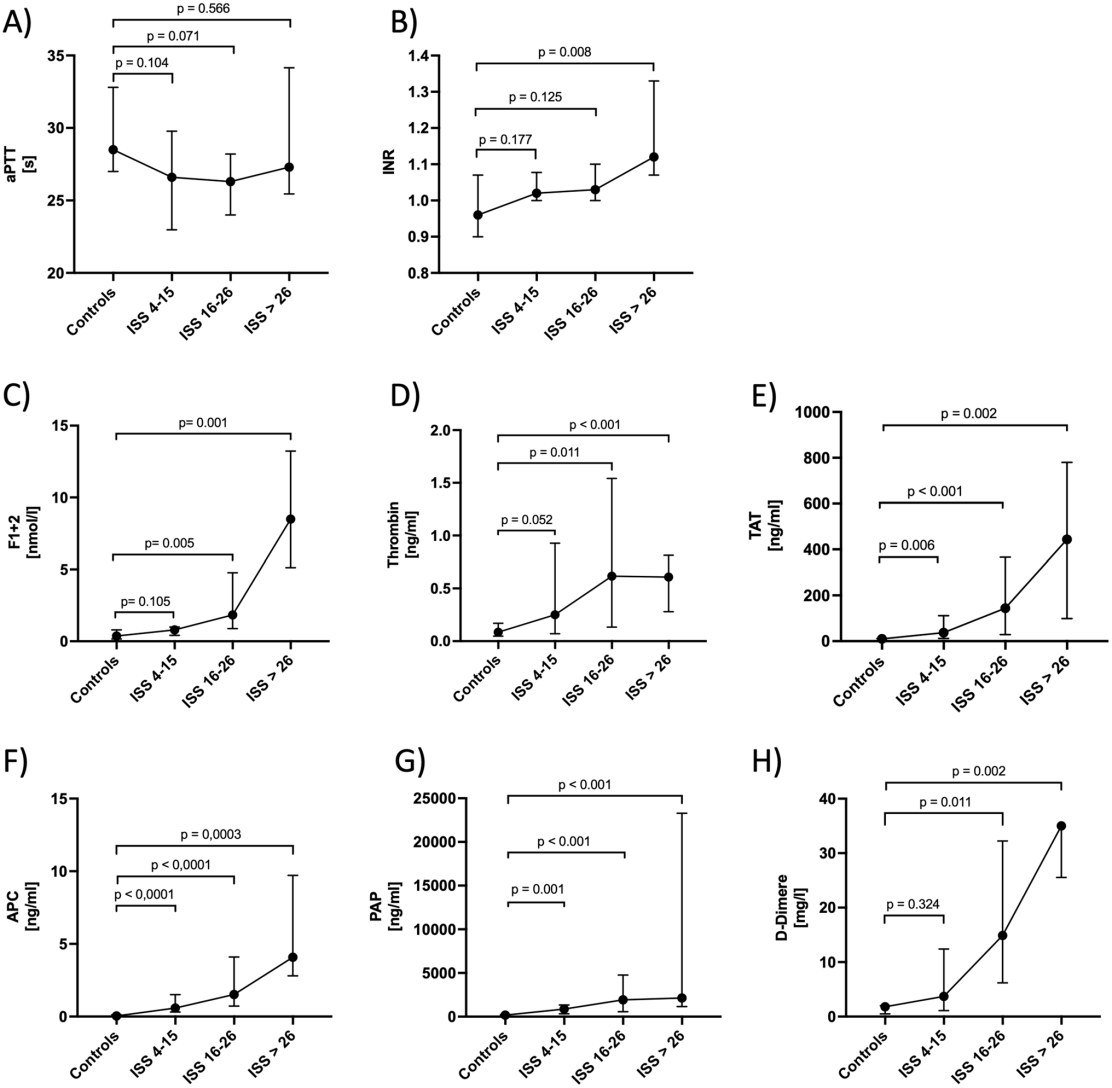
The analytes’ characteristics depending on the ISS are displayed. Not injured patients were taken as controls. For each group, a median and the 0.95 interval are shown. Results were considered as significant if *p* values were *p* < 0.05. *ISS* injury severity score, *aPTT* activated partial thromboplastin time, *INR* International normalized ratio, *F1* + *2* prothrombin fragments 1 and 2, *TAT* thrombin–antithrombin complex, *APC* activated protein C, *PAP* plasmin–antiplasmin complexes

Regarding underlying injury pattern, based on the major injured body part analyzed for patients with an AIS ≥ 3 in different body regions, no loco-regional distribution dependency could be observed for the global coagulation markers aPTT and INR (Fig. 2A, B). While the same was true for plasma levels of active thrombin (Fig. 2D), significant differences between affected or non-affected patients were found for the indirect thrombin markers F1 + 2 and TAT as well as D-Dimer (Fig. 2C, E, H). Interestingly, in the present cohort, patients with major injuries of the thorax or extremities/pelvis had significantly higher APC levels compared to those with only minor injured body regions, while patients suffering primarily from isolated TBI showed no such difference (Fig. 2F). Regarding PAP, only patients with severe injuries of the thoracic region showed significantly higher circulating levels than those with AIS < 3 (Fig. 2G), indicating a dependency of the loco-regional injury pattern.

### Plasma levels of hemostatic biomarkers vs. clinical bleeding parameters

**Fig. 3:**
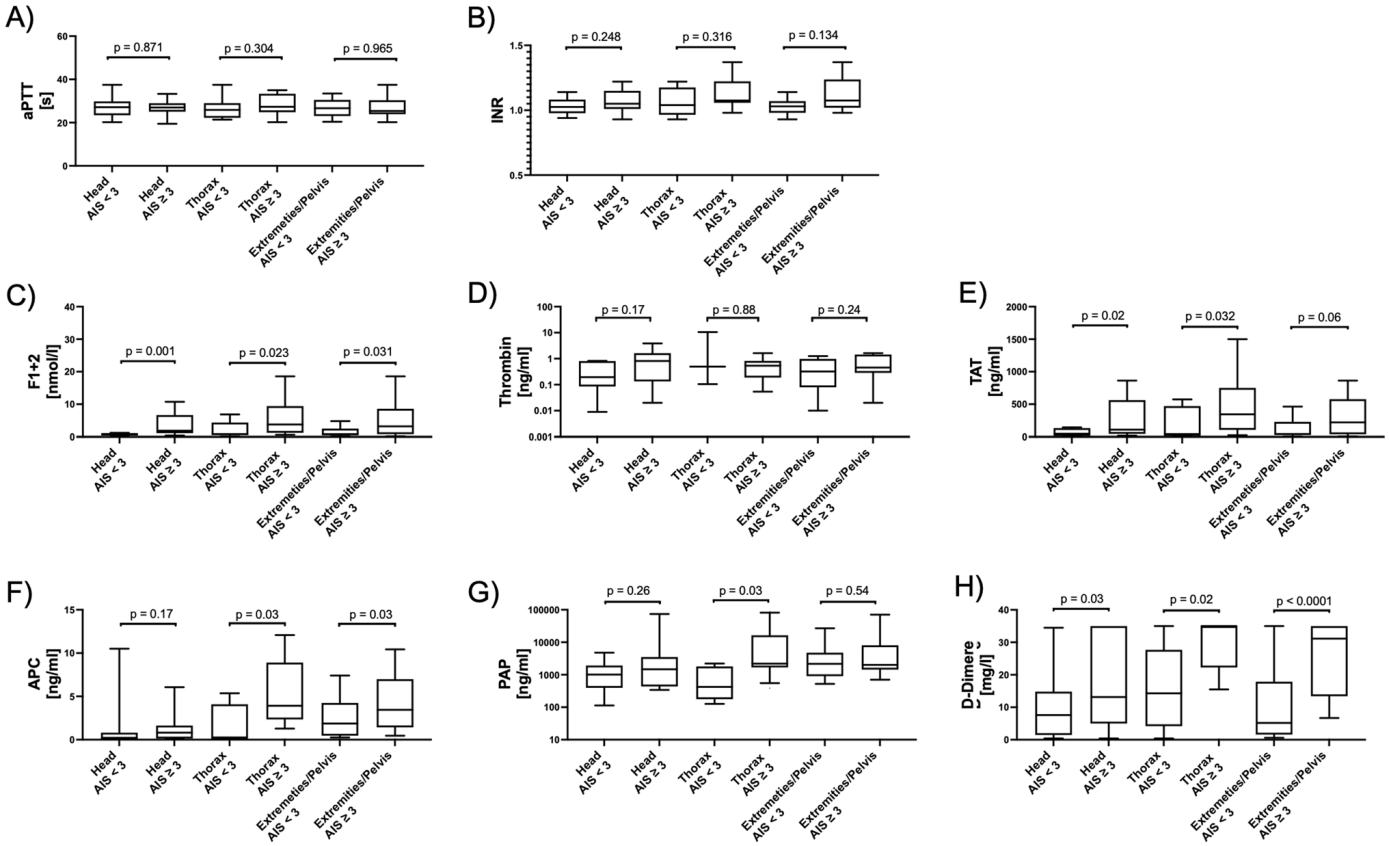
The analytes’ characteristics depending on the leading injury pattern (head, thorax, pelvis/extremities) are presented**.** Patients were considered as major injured if AIS ≥ 3. Differences between the groups as significant if *p* values were *p* < 0.05. *AIS* abbreviated injury scale, *aPTT* activated partial thromboplastin time, *INR* International normalized ratio, *F1* + *2* prothrombin fragments 1 and 2, *TAT* thrombin–antithrombin complex, *APC* activated protein C, *PAP* plasmin–antiplasmin complexes

For those patients defined as being “coagulopathic” (calculated TASH score ≥ 15 or a transfusion requirement within the first 24 h after admission or with clinical bleeding signs on admission), significantly higher plasma levels of F1 + 2, TAT, APC, PAP and D-Dimer were measured compared to “non coagulopathic” patients (Fig. 3 C, E, F–H). In contrast, no significant differences between “coagulopathic” and “non coagulopathic” patients were observed when analyzing aPTT, INR or plasma levels of active thrombin (Fig. 3A, B, D).

### Prognostic potential of hemostatic biomarkers to predict TIC

**Fig. 4:**
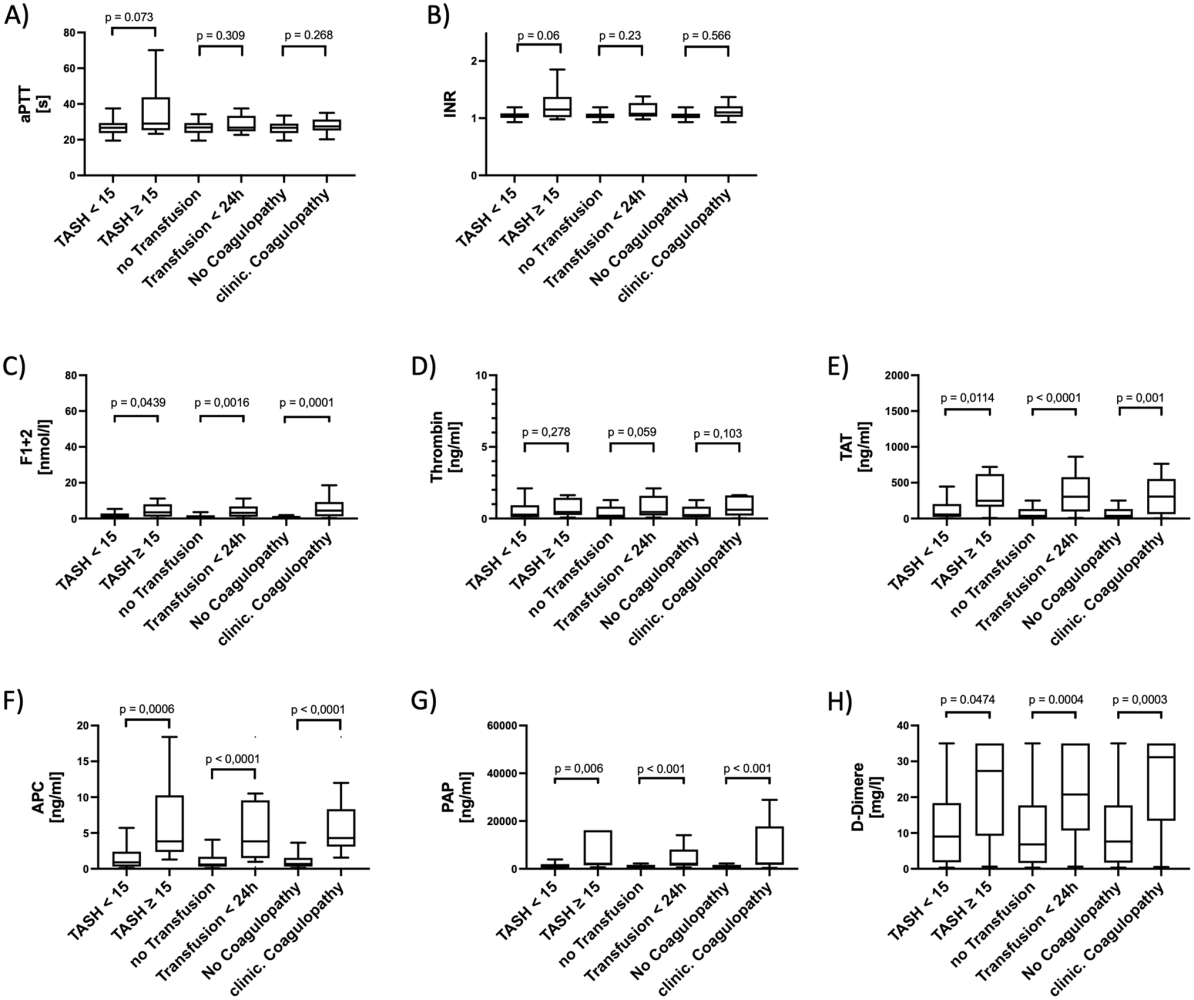
The analytes’ characteristics depending on a calculated or clinical bleeding tendency. Patients were dichotomized whether a bleeding tendency due to an underlying coagulopathic state could be presumed. The TASH (trauma-associated severe hemorrhage) Score is validated to assess whether a massive transfusion after trauma due to an underlying coagulopathy has a likelihood ≥ 50% after trauma (TASH score ≥ 15). As further surrogate markers for a clinically relevant bleeding tendency, we defined a transfusion of blood products with red blood cell concentrates (RBC) within the first 24 h after admission and an estimation of our clinical team whether they expected a bleeding phenotype within the trauma management based either on thromboelastometry, their experience or clinical signs of a bleeding tendency (e.g., prolonged bleeding after punctures, spontaneous or prolonged bleeding). For each value, a median and the 0.95 interval is displayed. Results were considered as significant if *p* values were *p* < 0.05. *TASH* trauma-associated severe hemorrhage, *aPTT* activated partial thromboplastin time, *INR* International normalized ratio, *F1* + *2* prothrombin fragments 1 and 2, *TAT* thrombin–antithrombin complex, *APC* activated protein C, *PAP* plasmin–antiplasmin complexes

To evaluate the prognostic potential of hemostatic biomarkers as measured in plasma on admission to predict a forthcoming coagulopathy, cutoff values were calculated by applying ROC curve analysis on TASH scores, transfusion requirements, and signs of clinical coagulopathy (Fig. 4, Suppl. Figs. 5, 6). This analysis revealed a superior AUC-range of 0.83–0.88 for APC, followed by 0.77–0.81 for PAP, 0.75–0.79 for TAT, and of 0.70–0.73 for F1 + 2. Accordingly, approximated best discriminative cutoff values for APC to predict the risk of developing signs of coagulopathy had been calculated. APC values above 1.53–2.57 ng/ml were predictive with a sensitivity between 76.2 and 88.9% and a specificity between 71.4 and 87.3%. Patients with APC levels above 1.53 ng/ml showed significantly higher values in SOFA scores (OR 2.314, 95% CI 0.496–1.4; *p* < 0.0001), stayed longer on the ICU (OR 1.04; 95% CI 1.005–1.086; *p* = 0.03) and in hospital (OR 1.04, 95% CI 0.013–0.073, *p* = 0.009), and were on a ventilator for longer periods of time (OR 1.05; 95% CI 0.001–0.008; *p* = 0.05).

## Discussion

The intensive attention to TIC over the last years revealed a complex coagulopathy, resulting in different bleeding phenotypes [20]. Current evidence suggests an alteration of the thrombin- as well as the APC pathway as key effectors in the pathophysiology of TIC, leading to fibrinogen- and platelet consumption as well as (hyper-)fibrinolysis. However, as demonstrated in several clinical trials and consistent with the results of the data presented in this study, standard coagulation parameters (like INR or aPTT) do not reliably identify patients at risk for developing TIC [21]. In consequence, this leads to an inconsistent definition of TIC criteria making the diagnosis of TIC challenging [10]. Our data support the hypothesis that TIC development is triggered by a trauma-induced overwhelming thrombin formation followed by secondary consumption of platelets, procoagulant clotting factors, APC formation and hyperfibrinolysis.

The amount of generated thrombin following injury is a critical factor that determines the structure and stability of fibrin clots [22]. These characteristics of thrombin might build the basis of a valuable clinical parameter to stratify the severity of trauma impact already on admission, or potentially allowing a differentiation between coagulopathic patients and those with a developing bleeding tendency [23]. However, in contrast to circulating indirect markers of thrombin generation and inhibition, we could not prove any loco-regional distribution pattern, significantly higher levels in “coagulopathic” patients or distinct correlation to hypoperfusion or shock parameters for plasma levels of active thrombin. These findings reflect the short circulatory half-life of active thrombin (< 1 min) when compared to plasma levels of F1 + 2 or TAT [24]. On the one hand, remote from the site of the injury, thrombin is efficiently complexed by the serine protease inhibitor antithrombin, leading to inactive TAT [25]. On the other hand, thrombin binds to endothelial thrombomodulin, leading to activation of the anticoagulant PC pathway [26].

Thus, consistent with this thrombin-driven mechanism was the observation of increased, ISS-dependent APC plasma levels. These data are in line with several clinical studies that describe elevated APC levels following major trauma [27]. In addition, depending on the mainly affected body regions, APC levels showed a region-dependent distribution. After severe thoracic trauma and injuries of the extremities going along with wide tissue damage, significantly higher APC levels were observed compared to patients showing an AIS < 3 in the respective region.

As a fibrinolytic marker, also PAP values showed a dependence on injury severity. Interestingly, especially after thoracic trauma and especially lung contusions, we observed significantly higher PAP values, being in line with the expected high fibrinolytic activity of lung tissue.

There are recent in vitro studies that question the regulatory role of APC in trauma as they demonstrated that platelet- and plasma FVa are resistant to APC cleavage at concentrations that had been reported for trauma patients [28]. Correlating APC with circulating extracellular histone levels, Kutcher and colleagues suggested a potential role of APC in mitigating the sterile inflammatory response after trauma through proteolysis of circulating histones [29]. However, our data show that APC levels were closely linked to a clinically bleeding tendency after trauma and that plasma APC levels might be used as marker of coagulation activation useful for the identification of patients with a poor prognosis.

Recent findings raised the question whether coagulopathy after TBI might differ from a systemic trauma population. After severe TBI, coagulopathy occurs in more than 60% of patients, but is widely uncommon in mild head injuries with a Glasgow Coma Scale (GCS) ≥ 13 (< 1%) [30]. Our data suggest that APC plasma levels do not correlate with TBI severity alone and consistent results were found for PAP as a marker of fibrinolytic activity. However, TBI-related coagulopathy seems to be more severe in patients with acidosis and high lactate concentrations, and hypoperfusion has been associated with an increased risk of hyperfibrinolysis via activation of the protein C pathway [31, 32]. Even though our cohort also consisted of patients with high injury severity, our data are lacking patients with circulatory shock on admission that might influence results.

Our data provide evidence for ISS-dependent APC generation depending on the major localization and the extent of the trauma. Indeed, it could be previously demonstrated that plasma levels of APC reliably reflect the activation status of the blood coagulation system [18] [33]. This can be ascribed to the kinetics (turnover) of APC generation by the thrombin–thrombomodulin complex and the relatively long circulatory half-life of the active enzyme of approx. 20 min [18, 26]. Based on these characteristics, elevated APC plasma levels on admission were found to be an independent predictor of mortality in patients with septic shock [34]. In the present study, when compared to other hemostatic biomarkers as done by ROC curve analysis, APC was identified as candidate marker to identify patients at risk for TIC.

Based on the present data, we cannot elucidate whether the observed derangement of the coagulation system might be the attempt to maintain perfusion in the face of tissue injury and shock or represent the result of a maximal effort to stop bleeding, leading to coagulation failure through consumption, dilution and/or loss of autoregulation. While it remains clear that coagulation disorders after trauma are widely heterogeneous, identification of major drivers and biomarkers is important to achieve a better understanding of the pathogenesis and to aim for better diagnostic tools to get closer to precise definition criteria of TIC. Hemostatic biomarkers to identify patients at risk in addition to the clinical picture might offer possibilities to initiate a targeted therapy earlier and to be included in risk stratification algorithms.

In this context, further studies are needed to confirm the predictive value of APC plasma levels as described above.

### Limitations

This study sought to analyze the role of hemostatic biomarkers, especially APC and thrombin, in the development of TIC. However, surrogate markers of endothelial function that is important for activation of the PC pathway were not studied. Furthermore, the identification of coagulopathic patients after trauma or patients at risk for TIC is challenging and could only be performed using surrogate markers such as TASH score, transfusion rate and clinical ratings.

In addition, patients who died in the early phase after trauma had to be excluded from the study due to our ethical approval and biased our mortality. In combination with the limited number of individuals included in our study combined with the heterogeneity of observed injury pattern, these imponderables may limit the generalizability of our results.

## Conclusion

Trauma-induced coagulopathy remains a heterogeneous syndrome whereby the underlying mechanisms are still poorly understood. We demonstrated that plasma levels of APC correlate with injury severity and trauma location and may serve as biomarker to identify patients at risk for TIC at admission.

## Supplementary Information

Below is the link to the electronic supplementary material.

Supplementary file1 To evaluate the prognostic potential of hemostatic biomarkers as measured in plasma on admission to predict a forthcoming coagulopathy, cut-off values were calculated by applying ROC curve analysis on TASH scores (Fig. 4), transfusion requirements (Supp. Fig. 5), and signs of clinical coagulopathy (Supp. Fig.6). (PNG 600 kb)

Supplementary file2 To evaluate the prognostic potential of hemostatic biomarkers as measured in plasma on admission to predict a forthcoming coagulopathy, cut-off values were calculated by applying ROC curve analysis on TASH scores (Fig. 4), transfusion requirements (Supp. Fig. 5), and signs of clinical coagulopathy (Supp. Fig.6). (PNG 685 kb)

Supplementary file3 To evaluate the prognostic potential of hemostatic biomarkers as measured in plasma on admission to predict a forthcoming coagulopathy, cut-off values were calculated by applying ROC curve analysis on TASH scores (Fig. 4), transfusion requirements (Supp. Fig. 5), and signs of clinical coagulopathy (Supp. Fig.6). (PNG 686 kb)

Supp. Figure 8:Correlation of APC values with standard coagulation values (INR) and shock parameters (Shock Index, Lactate, Base Excess). Classes for Base Excess were built using the ATLS-Shock-Index classification. For each item the correlation coefficient and a p-value is given.

Supp. Figure 9:Correlation of Thrombin values with standard coagulation values (INR) and shock parameters (Shock Index, Lactate, Base Excess). Classes for Base Excess were built using the ATLS-Shock-Index classification. For each item the correlation coefficient and a p-value is given.

## Data Availability

The datasets used and/or analyzed during the current study are available from the corresponding author on reasonable request.

## Abbreviations

AIS: Abbreviated injury scale
APC: Activated protein C
aPTT: Activated partial thromboplastin time
ATLS: Advanced trauma life support^®^
AUC: Area under the curve
CK: Creatine kinase
CMMC: Cologne-merheim medical centre
DIC: Disseminated intravascular coagulopathy
F1 + 2: Prothrombin activation fragment F1.2
FVa: Activated factors V
FV: Factor V
FVIIIa: Activated factors VIII
GCS: Glasgow coma scale
INR: International normalized ratio
ISS: Injury severity score
OECA: Oligonucleotide-based enzyme capture assay
OR: Odds ratio
PAI-1: Plasminogen activator inhibitor-1
PAP: Plasmin-antiplasmin complexes
PC: Protein C
RBC: Red blood cells
ROC: Receiver-operating characteristic
SOFA: Sepsis-related organ failure assessment
TAFI: Thrombin-activatable fibrinolysis inhibitor
TASH: Trauma associated severe hemorrhage
TAT: Thrombin-antithrombin complexes
TBI: Traumatic brain injury
TIC: Trauma-induced coagulopathy
TM: Thrombomodulin
t-PA: Tissue-type plasminogen activator
