# Supplementary figures and images for: Plasmatic coagulation profile after major traumatic injury: a prospective observational study
Source: Eur J Trauma Emerg Surg. 2022 May 16;48(6):4595–606. doi: 10.1007/s00068-022-01971-6 (PMC9712322; doi:10.1007/s00068-022-01971-6)

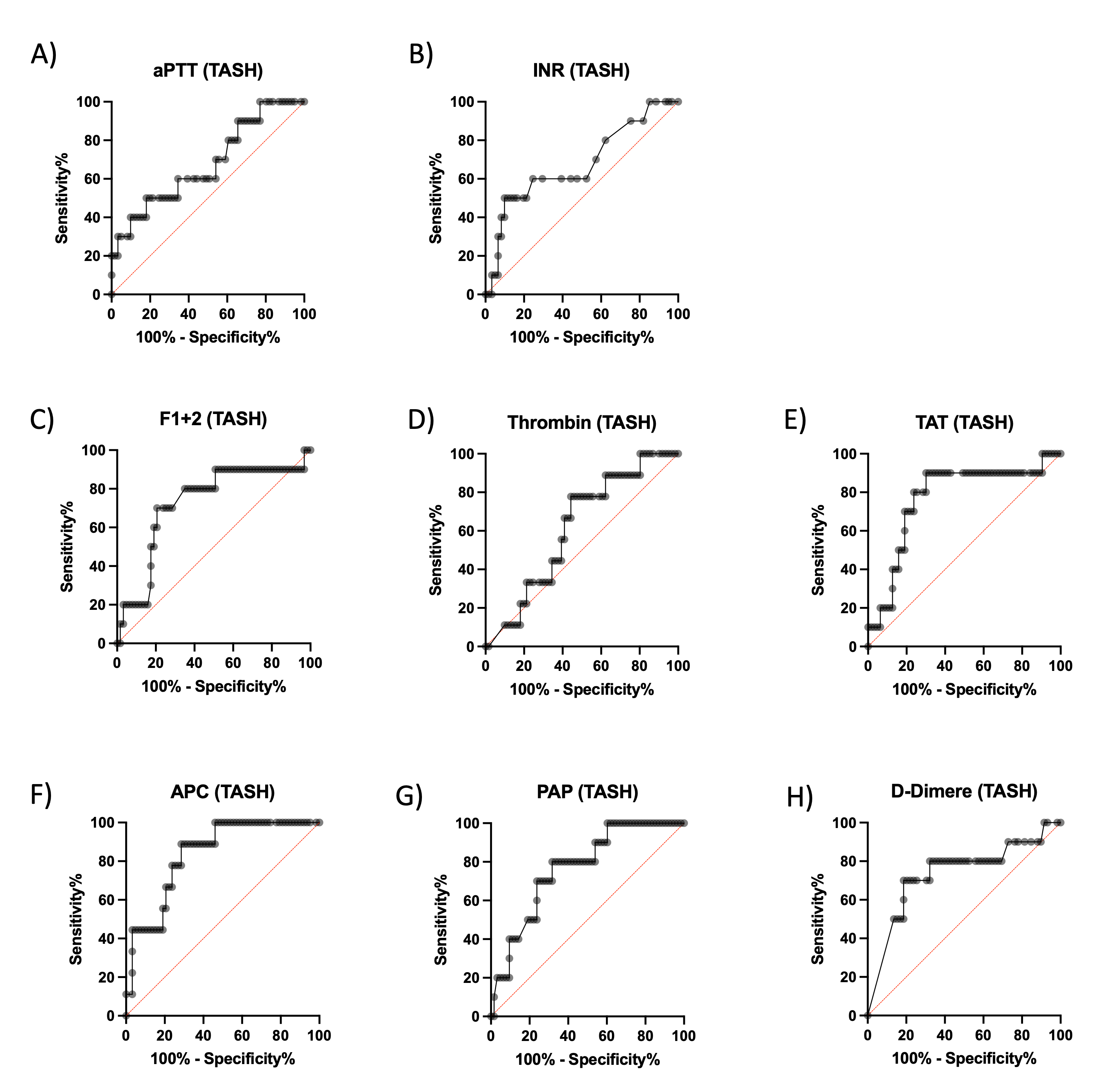

Supplement: Supplementary file 1 — Supplementary file1 To evaluate the prognostic potential of hemostatic biomarkers as measured in plasma on admission to predict a forthcoming coagulopathy, cut-off values were calculated by applying ROC curve analysis on TASH scores (Fig. 4), transfusion requirements (Supp. Fig. 5), and signs of clinical coagulopathy (Supp. Fig.6). (PNG 600 kb) [file 68_2022_1971_MOESM1_ESM.png]

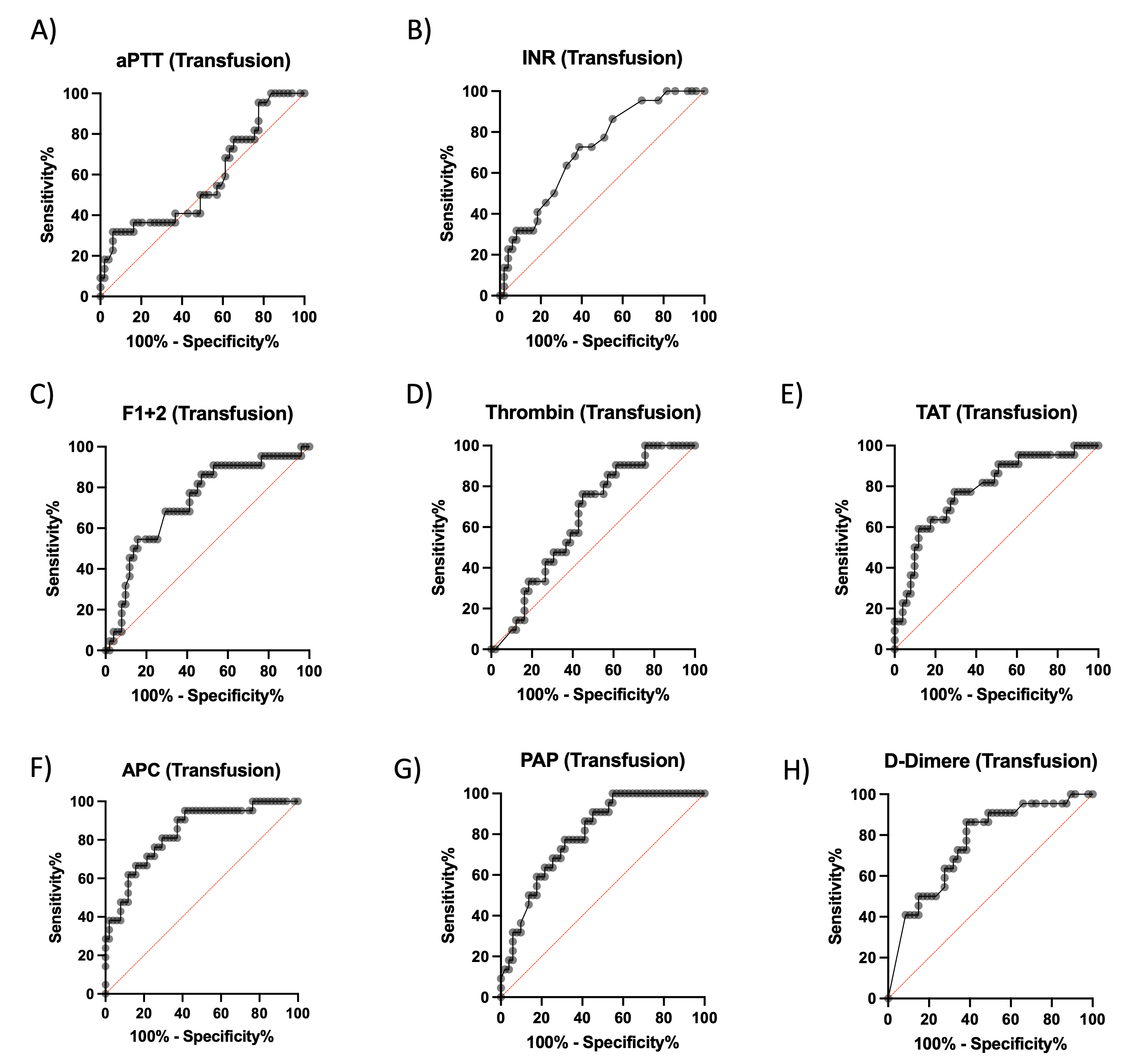

Supplement: Supplementary file 2 — Supplementary file2 To evaluate the prognostic potential of hemostatic biomarkers as measured in plasma on admission to predict a forthcoming coagulopathy, cut-off values were calculated by applying ROC curve analysis on TASH scores (Fig. 4), transfusion requirements (Supp. Fig. 5), and signs of clinical coagulopathy (Supp. Fig.6). (PNG 685 kb) [file 68_2022_1971_MOESM2_ESM.png]

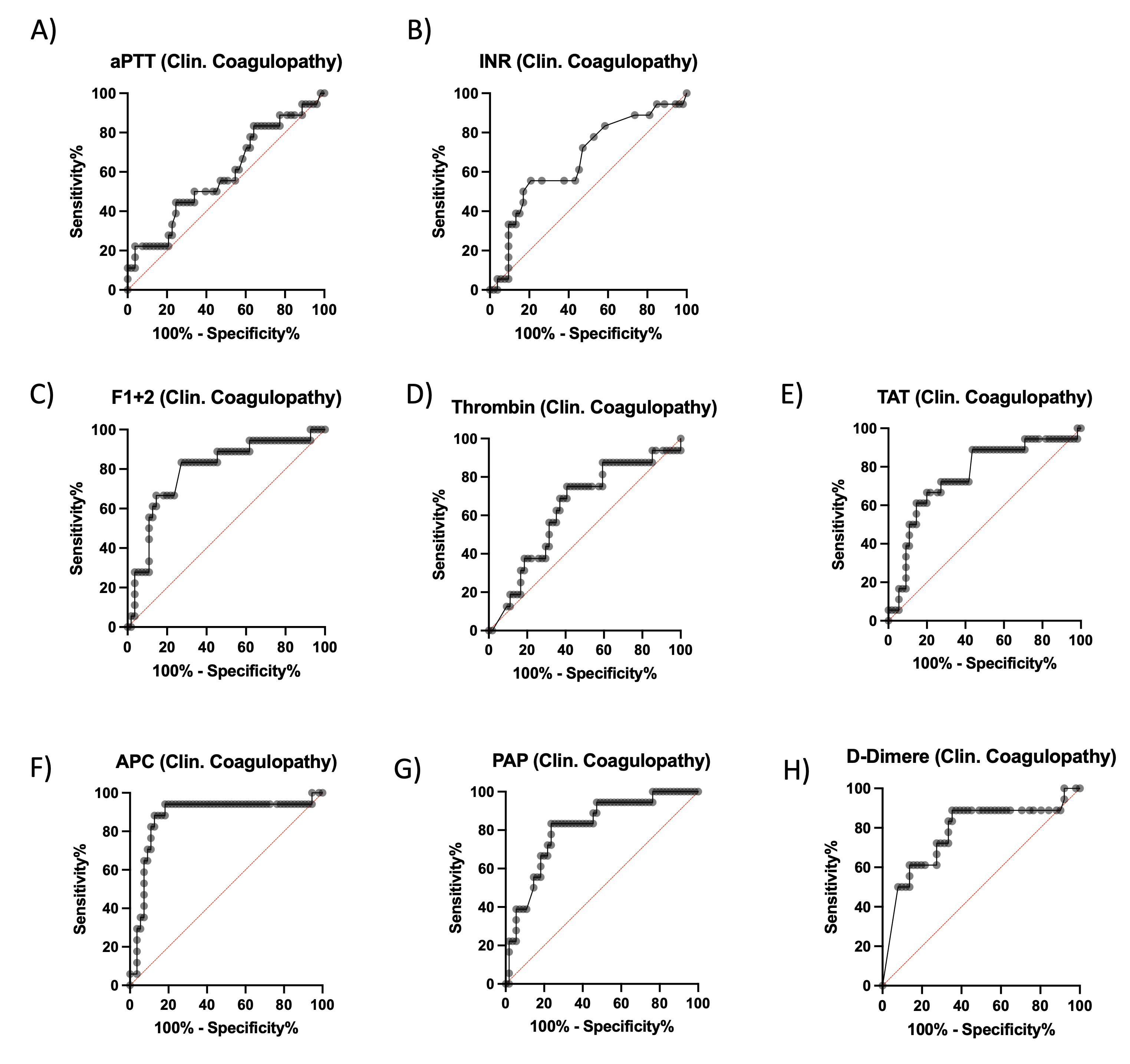

Supplement: Supplementary file 3 — Supplementary file3 To evaluate the prognostic potential of hemostatic biomarkers as measured in plasma on admission to predict a forthcoming coagulopathy, cut-off values were calculated by applying ROC curve analysis on TASH scores (Fig. 4), transfusion requirements (Supp. Fig. 5), and signs of clinical coagulopathy (Supp. Fig.6). (PNG 686 kb) [file 68_2022_1971_MOESM3_ESM.png]

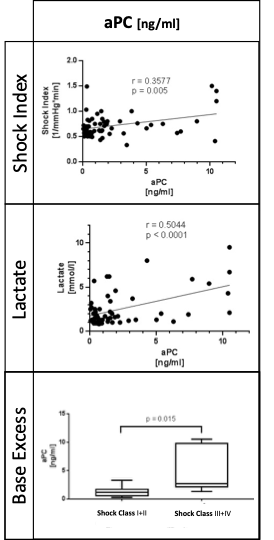

Supplement: Supplementary file 4 — Supp. Figure 8:Correlation of APC values with standard coagulation values (INR) and shock parameters (Shock Index, Lactate, Base Excess). Classes for Base Excess were built using the ATLS-Shock-Index classification. For each item the correlation coefficient and a p-value is given. [file 68_2022_1971_MOESM4_ESM.png]

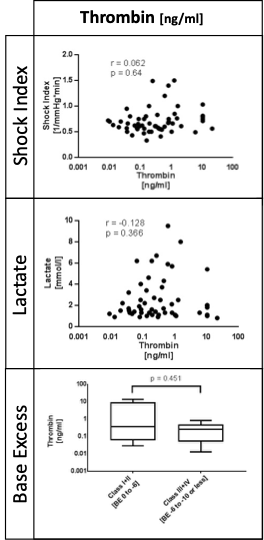

Supplement: Supplementary file 5 — Supp. Figure 9:Correlation of Thrombin values with standard coagulation values (INR) and shock parameters (Shock Index, Lactate, Base Excess). Classes for Base Excess were built using the ATLS-Shock-Index classification. For each item the correlation coefficient and a p-value is given. [file 68_2022_1971_MOESM5_ESM.png]
